# Seronegative Rheumatoid Arthritis: A Distinct Immunopathological Entity with Erosive Potential

**DOI:** 10.3390/medsci14010014

**Published:** 2025-12-28

**Authors:** Florent Lhotellerie, Ala Eddine Ben Ismail, Julie Sarrand, Muhammad Soyfoo

**Affiliations:** Department of Rheumatology, Hôpital Universitaire de Bruxelles, Université libre de Bruxelles, 1070 Brussels, Belgium; lhotellerie.florent@gmail.com (F.L.); alaeldine1@outlook.fr (A.E.B.I.); julie.sarrand@ulb.be (J.S.)

**Keywords:** rheumatoid arthritis, seronegative, autoantibody-negative, ACPA, rheumatoid factor, anti-modified protein antibodies, AMPA, anti-carbamylated protein antibodies erosive disease, disease-modifying antirheumatic drugs, precision medicine

## Abstract

**Background:** Seronegative rheumatoid arthritis (SNRA), defined by the absence of rheumatoid factor (RF) and anti-citrullinated peptide antibodies (ACPA), represents 20–30% of rheumatoid arthritis cases. Once considered a milder phenotype, SNRA is now recognised as a heterogeneous entity in which a substantial subset of patients develops structural progression comparable to seropositive RA. The binary RF/ACPA-based definition is increasingly viewed as insufficient, as the broader anti-modified protein antibody (AMPA) family—including antibodies against carbamylated, acetylated and malondialdehyde–acetaldehyde–modified proteins—indicates that many “seronegative” patients may harbour unconventional humoral autoimmunity undetected by standard assays. **Objectives:** To synthesise contemporary insights into the epidemiology, immunopathology, diagnostic challenges and therapeutic management of SNRA, with emphasis on erosive versus non-erosive phenotypes and the implications of the AMPA paradigm. **Methods:** A comprehensive literature search of PubMed, Cochrane Library and Google Scholar identified randomised trials, observational cohorts and systematic reviews, with focus on studies published within the past decade. Results: SNRA displays partially distinct immune features, including lower formation of tertiary lymphoid structures and variable activation of innate inflammatory circuits. However, the traditional adaptive–versus–innate dichotomy is overly reductionist. Growing evidence suggests that unconventional humoral responses directed against non-classical post-translational modifications may be present in a proportion of RF/ACPA-negative patients. Additional qualitative dimensions—such as IgA isotypes and fine-specificity profiles—represent further heterogeneity with potential prognostic significance. Although ACPA remains the strongest predictor of erosive progression, up to one-third of seronegative patients develop erosions within five years. The 2010 ACR/EULAR criteria may delay diagnosis in SNRA. Cytokine inhibitors and JAK inhibitors show largely serostatus-independent efficacy, whereas B-cell and T-cell–targeted therapies demonstrate attenuated responses in SNRA. **Conclusions:** SNRA is clinically and immunologically diverse. Integrating the AMPA framework is essential for refining classification and prognostication. Distinguishing erosive from non-erosive forms may guide treatment, while future work should prioritise biomarkers predicting progression and therapeutic response.

## 1. Introduction

Rheumatoid arthritis (RA) is a chronic autoimmune inflammatory disease primarily targeting peripheral joints. It presents as symmetrical, progressive synovitis that can lead to cartilage degradation, bone erosion, and ultimately functional disability. Its prevalence is highest in Western populations, surpassing 300 cases per 100,000 inhabitants [1]. In Northern Europe, prevalence approaches 0.4%, with an annual incidence of 20–30 new cases per 100,000 person-years. Women are disproportionately affected (female-to-male ratio 2–3:1), and onset typically occurs around the sixth decade of life. Approximately 20–30% of individuals with RA exhibit a seronegative phenotype, defined by the absence of rheumatoid factor (RF) and anti-cyclic citrullinated peptide antibodies (ACPA) [2,3,4]. Traditionally regarded as a milder variant with a lower risk of structural progression, this subgroup has long been considered clinically and prognostically distinct. It is important to acknowledge at the outset that the term ‘seronegative RA’ as conventionally used refers specifically to RF/ACPA-negative disease and should not be equated with ‘truly autoantibody-negative’ or ‘immunonegative’ RA. Over the past decade, substantial evidence has accumulated demonstrating that a significant proportion of patients classified as seronegative by standard criteria harbour autoantibodies directed against a broader spectrum of anti-modified protein antibodies (AMPA), including anti-carbamylated protein (anti-CarP), anti-acetylated protein, and anti-malondialdehyde–acetaldehyde (MAA)-modified protein antibodies [5]. This conceptual distinction is critical for interpreting the clinical and immunological heterogeneity observed within the seronegative spectrum and has direct implications for biomarker development, prognostic stratification, and therapeutic decision-making.

Compelling evidence over recent decades has challenged this assumption. Bone erosion—central to structural damage and long-term disability—also occurs in seronegative RA. These lesions, detectable at baseline or emerging during follow-up, arise from synovial pannus infiltration by inflammatory cells driving osteoclast activation and bone resorption, thereby contributing substantially to functional decline [6]. Imaging modalities such as conventional radiography, musculoskeletal ultrasound, and magnetic resonance imaging (MRI) allow sensitive detection of these lesions. Among available scoring systems, the van der Heijde–modified Sharp score (vdH-mTSS) remains the most widely used and sensitive for quantifying erosion and joint-space narrowing [7,8].

Seronegative patients also differ from seropositive counterparts in their extra-articular profile. Manifestations such as interstitial lung disease (ILD) occur less frequently and are more strongly linked to ACPA positivity [9,10]. Epidemiological data further reveal a relative rise in seronegative forms over recent years, contrasting with a decline in seropositive disease. This shift—likely influenced by demographic aging and evolving environmental exposures—suggests a transformation of the underlying immunological architecture of RA and raises important questions about optimising management in seronegative RA (SNRA) [11,12].

Despite this evolution, the literature remains surprisingly sparse regarding the therapeutic distinction between erosive and non-erosive SNRA. This gap limits our ability to design personalised treatment strategies and to anticipate divergent disease trajectories within this heterogeneous population. Better characterisation of these subgroups is essential to identify patients at risk of structural progression and to distinguish them from those who remain non-erosive despite ongoing symptoms.

This review aims to clarify the complex heterogeneity of SNRA by synthesising knowledge accumulated primarily over the last decade. It examines epidemiology, pathophysiology, classification challenges, differential diagnosis, and treatment responses in predominantly seronegative forms, with particular attention to the distinction between erosive and non-erosive phenotypes. Critically, we address the conceptual limitations of the binary seropositive/seronegative framework and integrate recent advances in understanding unconventional humoral autoimmunity within the AMPA spectrum.

## 2. Pathophysiology

### 2.1. Environmental Factors and Genetic Susceptibility

Seropositive rheumatoid arthritis (SPRA) is strongly influenced by genetic factors, particularly HLA-DRB1*01, *04, and *DQ8 alleles, which account for approximately 50% of the genetic susceptibility and are associated with an increased risk of severe bone erosions. In contrast, seronegative RA (SNRA) appears less dependent on this genetic predisposition and more influenced by environmental determinants. Smoking, bacterial infections (e.g., *Porphyromonas gingivalis*, *Aggregatibacter actinomycetemcomitans*), and lifestyle factors such as diet, physical activity, or alcohol consumption play notable roles in disease initiation [13,14].

#### 2.1.1. Lifestyle Factors

Lifestyle factors play notable roles in disease initiation in both seropositive and seronegative RA. Smoking represents a well-established risk factor, with particularly strong associations with ACPA-positive disease, though effects on seronegative RA are also documented. Diet, physical activity, alcohol consumption, and obesity have been variably associated with RA risk, though their relative contributions to seropositive versus seronegative disease require further characterization. A recent meta-analysis demonstrated that silica exposure was associated with both ACPA-positive and ACPA-negative RA to a similar extent [11].

#### 2.1.2. Infectious Triggers and the Oral–Systemic Axis

Bacterial infections, particularly those associated with periodontal disease, have emerged as important contributors to RA pathogenesis and warrant detailed consideration separate from general lifestyle factors. *Porphyromonas gingivalis* and *Aggregatibacter actinomycetemcomitans* are the most extensively studied periodontal pathogens in relation to RA. *P. gingivalis* is unique among prokaryotes in expressing a peptidylarginine deiminase (PPAD) capable of citrullinating host proteins, thereby generating neoepitopes that may trigger ACPA production. *A. actinomycetemcomitans* produces leukotoxin A (LtxA), which induces hypercitrullination in neutrophils through pore formation and calcium influx, providing an alternative pathway to citrullinated antigen generation [13].

Beyond *P. gingivalis* and *A. actinomycetemcomitans*, other oral pathogens including Filifactor alocis have been implicated in RA immunopathology. These bacterial species express immunomodulatory toxins and release outer membrane vesicles (OMVs) containing virulence factors that can translocate systemically. Importantly, these vesicles are small enough to cross biological barriers, including the blood–brain barrier, potentially explaining the systemic inflammatory effects of localized periodontal infection. The ability of bacterial OMVs to carry citrullinated antigens or citrullinating enzymes systemically may contribute to the initiation of autoimmunity at sites distant from the oral cavity [12,14].

The immunomodulatory effects of chronic infections may induce variable host responses, and it remains an open question whether ACPA and RF in some patients represent responses to external microbial antigens without direct connection to joint inflammation. Data from pre-RA cohorts demonstrate that autoantibodies can be detected years before clinical disease onset, suggesting that breaks in tolerance may occur at mucosal sites (oral, lung, gut) before synovial involvement. This ‘mucosal origins’ hypothesis is particularly relevant to understanding the heterogeneity of SNRA, as patients lacking classical autoantibodies may have been exposed to different antigenic triggers or may mount qualitatively different immune responses to similar exposures.

### 2.2. MicroRNAs and Post-Transcriptional Regulation

Recent studies have highlighted the role of microRNAs (miRNAs) in RA pathogenesis. In particular, functional variants of miRNA-499 and miRNA-146 are abnormally expressed in cells implicated in the disease process. These miRNAs influence the regulation of key genes involved in inflammation, cellular differentiation, and autoimmunity. Their dysregulation may contribute to explaining certain forms of susceptibility to RA [15,16].

### 2.3. Predominant Innate Immune Response in Seronegative RA

**Figure 1:**
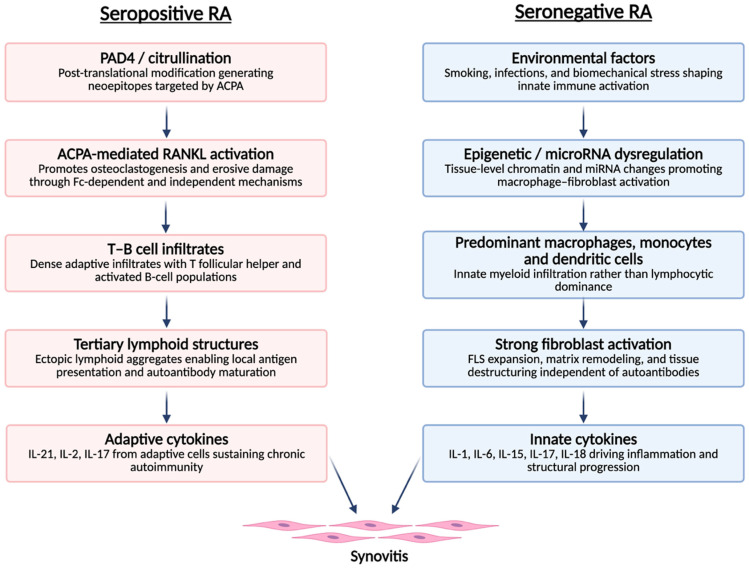
Distinct immunopathological pathways leading to synovitis in seropositive and seronegative rheumatoid arthritis. Seropositive RA follows an adaptive-immune pathway initiated by PAD4-mediated citrullination and ACPA-driven osteoclastogenic signalling, leading to T–B cell infiltrates, tertiary lymphoid structures and adaptive cytokine production. Seronegative RA is dominated by an innate–stromal pathway involving environmental triggers, epigenetic dysregulation, myeloid-cell infiltration, fibroblast activation and innate cytokines. Both pathways converge on a shared synovial inflammation phenotype. ACPA: anti-citrullinated protein antibodies; FLS: fibroblast-like synoviocytes; IL: interleukin; miRNA: microRNA; PAD4: peptidylarginine deiminase 4 refs [17,18,19,20,21,22].

SNRA seems to be characterized by distinct immunopathological mechanisms compared with SPRA (Figure 1). Recent evidence has highlighted a predominance of innate immune responses in seronegative forms, in contrast to the adaptive immune activation typical of seropositive disease. Spatial transcriptomic analyses of synovitis in these patients reveal increased infiltration of dendritic cells, together with an absence of tertiary lymphoid structures, whereas seropositive forms exhibit lymphoplasmacytic infiltrates rich in T and B lymphocytes. Moreover, macrophages and monocytes predominate in the synovial tissue of seronegative patients, suggesting a distinct and potentially more heterogeneous pattern of immune activation [16,17]. The specific antigens responsible for attracting and activating immune cells in SNRA remain incompletely characterized. In seropositive disease, citrullinated proteins—including fibrinogen, vimentin, α-enolase, and type II collagen—serve as immunodominant targets. In seronegative disease, the antigenic landscape appears more heterogeneous. Potential candidate antigens include: [1] carbamylated proteins generated through non-enzymatic modification of lysine residues; [2] acetylated proteins; [3] oxidatively modified proteins including MAA adducts; [4] bacterial antigens from periodontal or gut pathogens that may cross-react with joint proteins; and [5] stress-induced neoepitopes on synovial fibroblasts or chondrocytes. The relative contribution of each antigenic class to immune cell recruitment and activation in SNRA subsets requires further investigation [18,19,20].

However, it is important to avoid an overly rigid adaptive-versus-innate dichotomy. Emerging evidence indicates that humoral autoimmunity in SNRA is not absent but frequently unconventional, heterogeneous, and directed against non-classical post-translational modifications (PTMs). The detection of anti-CarP, anti-acetylated protein, and other AMPA in subsets of RF/ACPA-negative patients challenges the notion that SNRA is fundamentally ‘non-autoimmune.’ Rather, these findings suggest that B-cell-mediated processes may operate through distinct antigenic targets not captured by standard serological testing [18,19,20,21,22]. The interactions between unconventional autoantibody responses and innate immune pathways—including macrophage activation via immune complex formation—represent an important area for further investigation and may help explain the clinical and structural heterogeneity observed within the seronegative spectrum (Figure 1).

### 2.4. Central Role of Synovial Fibroblasts

Synovial fibroblasts play a pivotal role in joint destruction, independent of the presence of specific autoantibodies. In RA, these cells acquire an aggressive phenotype, characterized by increased secretion of matrix metalloproteinases, proinflammatory cytokines such as TNF-α, and osteolytic mediators. This activation contributes to osteoclast differentiation and bone resorption, even in the absence of ACPA or RF, underscoring their role in SNRA [17].

## 3. Non-Conventional Autoantibodies, the AMPA Framework and Post-Translational Modifications

Although patients with seronegative RA (SNRA) are, by definition, negative for RF and ACPA, several non-conventional autoantibodies have been identified and may contribute to disease pathophysiology. The recognition that autoimmunity in RA extends beyond citrullination to encompass a broader spectrum of post-translational modifications (PTMs) has given rise to the anti-modified protein antibody (AMPA) concept. This framework fundamentally challenges the binary seropositive/seronegative classification by demonstrating that a substantial proportion of conventionally defined ‘seronegative’ patients harbour autoantibodies against carbamylated, acetylated, and other modified self-proteins [5].

### 3.1. Citrullination: Endogenous and Exogenous Pathways

Citrullination is catalyzed by peptidyl-arginine deiminase (PAD) enzymes, particularly PAD4, whose involvement in RA is well established. Proteins modified through citrullination—including α-enolase, fibrinogen, filaggrin, vimentin, and type II collagen—become immunogenic and trigger an autoimmune response via ACPA production. Importantly, citrullination can also be induced by oral pathogens through distinct mechanisms. *P. gingivalis* expresses a unique prokaryotic enzyme (PPAD) that directly promotes citrullination of bacterial and host proteins. *A. actinomycetemcomitans*, through its leukotoxin A, induces hypercitrullination in neutrophils by triggering calcium-dependent activation of endogenous PAD enzymes. These pathogen-driven citrullination pathways may play critical roles in breaking tolerance to modified self-proteins and initiating the autoimmune cascade in RA, potentially explaining differential autoantibody profiles between patients with and without periodontal disease.

Antibodies against PAD4 (anti-PAD4) have also been identified in RA patients. However, their diagnostic value remains limited: while their specificity reaches 82–100%, sensitivity ranges from 17% to 60%. Measurement of anti-PAD4, in addition to ACPA alone or in combination with RF, only marginally improves the diagnostic performance of RA [21,22].

### 3.2. Inflammasome Activation and Post-Translational Modifications

Linked to citrullination processes, activation of inflammasomes has emerged as an important mechanism connecting innate immunity to autoantibody generation in RA. The NLRP3 inflammasome, in particular, is activated by various danger signals present in the rheumatoid joint, including monosodium urate crystals, reactive oxygen species, and extracellular ATP. Upon activation, NLRP3 triggers caspase-1-mediated cleavage of pro-IL-1β and pro-IL-18, generating active cytokines that perpetuate inflammation.

Importantly, inflammasome activation can promote citrullination through several mechanisms: [1] neutrophil extracellular trap (NET) formation, which releases citrullinated histones and other proteins; [2] pyroptotic cell death, which exposes intracellular citrullinated antigens; and [3] amplification of PAD enzyme activity through calcium flux. The AIM2 inflammasome, activated by cytosolic DNA, may also contribute to RA pathogenesis, particularly in the context of NETosis. Recent studies suggest that inflammasome-driven IL-1β and IL-18 production synergizes with adaptive immune responses to amplify autoantibody production and promote erosive disease. Targeting inflammasome pathways may therefore represent a therapeutic strategy with relevance to both seropositive and seronegative RA.

### 3.3. Anti-Carbamylated Protein Antibodies and the AMPA Spectrum

Among non-conventional autoantibodies, anti-carbamylated protein antibodies (anti-CarP) have been particularly studied. They target proteins modified by carbamylation, a post-translational modification distinct from citrullination, in which lysine residues are converted into homocitrulline. Anti-CarP antibodies are present in 7–29% of double-seronegative patients, underscoring their potential to refine the classification of so-called seronegative forms. Recent data further indicate that anti-CarP responses extend beyond classical ACPA-related pathways: Dibrov et al., in a contemporary cohort, reported substantial anti-CarP positivity in both ACPA-positive and ACPA-negative RA, reinforcing the view that carbamylation-driven autoimmunity constitutes an additional layer of the humoral response. These antibodies appear independent of ACPA, without cross-reactivity, and may thus reflect complementary immune mechanisms. Their role in structural progression and synovial inflammation is currently under investigation [18,19,20].

Critically, an observational cohort study published in The Lancet Rheumatology demonstrated that autoantibodies directed against citrullinated, carbamylated, and acetylated peptides are independently associated with radiographic progression in patients with new-onset rheumatoid arthritis [22]. This finding is of particular relevance to SNRA, as it suggests that non-conventional AMPA may serve as biomarkers of erosive risk in patients who test negative for standard RF and ACPA. The independent association of each AMPA type with structural progression implies additive or complementary pathogenic mechanisms and highlights the potential clinical utility of extended autoantibody profiling in risk stratification.

Beyond these well-characterised autoantibodies, recent work has explored broader panels of non-conventional epitopes and post-translational modifications (PTMs) in an attempt to improve the detection of seronegative disease. A 2025 narrative review by Yi et al. highlighted rapid technological progress in multiplex immunoassays capable of simultaneously assessing citrullinated, carbamylated, and oxidatively modified antigens, thereby expanding the measurable autoantibody repertoire beyond traditional RF and ACPA. These platforms offer increased analytical sensitivity and capture a wider range of early breaks in tolerance associated with diverse PTMs such as carbamylation, malondialdehyde–acetaldehyde (MAA) adduct formation, and other oxidative neoepitopes. However, despite their conceptual relevance to SNRA, most validation cohorts in these studies remain heavily enriched in seropositive patients, limiting the generalisability of these findings to truly autoantibody-negative RA. To date, no combination of non-conventional autoantibodies or PTM-derived neoantigen signatures has demonstrated robust diagnostic performance at the individual level in seronegative patients. This reinforces the current dependence on RF/ACPA and imaging, and underscores the need for biomarker strategies specifically validated in seronegative RA [23,24,25].

### 3.4. Qualitative Features of Autoantibody Responses: Isotypes and Fine Specificity

Beyond binary autoantibody status, qualitative dimensions of the humoral response—including isotype distribution and fine specificity (FS) profiles—are increasingly recognized as key components of RA immunopathology. The predominance of IgA versus IgG isotypes, as well as IgG subclass distribution (particularly IgG1 and IgG4), may reflect important biological differences in disease initiation, mucosal involvement, epitope spreading, and effector mechanisms. IgA-ACPA and IgA-RF, for instance, have been associated with mucosal origins of autoimmunity and may identify patients with distinct environmental exposures or genetic backgrounds.

Several studies have demonstrated that autoantibody isotypes and subclass distribution influence inflammatory potential, complement activation, and clinical outcomes. Fine specificity profiling—assessing reactivity against multiple citrullinated or modified epitopes—provides additional prognostic information, as broader epitope recognition is generally associated with more aggressive disease. These qualitative features have also been linked to differential responses to targeted therapies, suggesting their potential contribution to future stratification strategies in precision medicine approaches.

Beyond their diagnostic and classificatory relevance, extended autoantibody profiling may also have therapeutic implications. Recent findings suggest that non-conventional autoantibodies could modulate treatment response and thus contribute to a more nuanced stratification of RA. In a study of abatacept-treated patients, Floris et al. evaluated anti-PAD4, anti-CarP and anti-RA33 antibodies alongside RF and ACPA, and reported that specific autoantibody constellations were associated with differential clinical responses to abatacept. Although these observations remain exploratory and require replication in larger, independent cohorts, they illustrate the potential clinical value of moving beyond a binary ‘seropositive versus seronegative’ framework. Richer autoantibody signatures—integrating classical and non-classical specificities—may ultimately support more personalized therapeutic strategies in RA, including within the seronegative spectrum [24,25,26,27].

## 4. Clinical Phenotypes and Structural Progression

Historically regarded as a milder form of RA, seronegative RA (SNRA) in fact displays substantial clinical and structural heterogeneity. Data from the French ESPOIR cohort illustrate that autoantibody negativity does not guarantee a benign course. At baseline, 18.9% of ACPA− patients already had radiographic erosions, compared with 31.2% of ACPA+ patients; after three years, these proportions increased to 29% and 63%, respectively [28]. Thus, while ACPA positivity is clearly associated with a substantially higher structural risk, a non-negligible proportion of seronegative patients nonetheless follow an erosive trajectory [29].

These findings underscore that SNRA may follow a destructive course, requiring rigorous management. Early identification of high-risk erosive forms is therefore a major clinical challenge.

Several clinical and biological factors have been identified as predictors of erosion development in seronegative patients: persistent inflammatory activity (high DAS28, elevated CRP and ESR) [27,28], longer symptom duration before treatment initiation [23], active smoking [30], presence of erosions at diagnosis [31], and male sex [32,33,34]. These factors highlight the importance of thorough baseline assessment and an adapted therapeutic strategy, even in the absence of autoantibodies.

A study by Nordberg et al. demonstrated significantly higher early inflammatory activity in seronegative patients compared with seropositive ones, with a median swollen joint count of 17 versus 8 (*p* < 0.001), along with higher ultrasound inflammatory scores [33]. However, over time, ACPA-positive patients tended to develop more severe disease, with higher numbers of swollen and tender joints and elevated DAS28 scores. Thus, while SNRA may initially present with high disease activity, autoantibody positivity remains a predictor of unfavorable structural progression [34,35,36,37]. These results highlight a potential dissociation between serological profile and inflammatory severity, emphasizing the need for complementary tools to guide management. The key distinctions between SNRA and Seropositive RA are represented in Table 1.

## 5. ACR/EULAR 2010 Classification: Limitations in Seronegative Forms

The 2010 ACR/EULAR classification criteria were developed to facilitate earlier identification of RA by incorporating several domains: number and type of joints involved, presence of inflammatory biomarkers (CRP/ESR), duration of joint symptoms, and the presence of autoantibodies (RF, ACPA) [30]. Serological positivity contributes substantially to the overall score, providing 2–3 points out of the 6 required for classification. This heavy weighting of serological markers favors the classification of seropositive forms but disadvantages seronegative patients, who must present with more extensive joint involvement—at least ten joints, including at least one small joint—to reach the diagnostic threshold [30,31].

### 5.1. Serology Weighting and Classification Bias

The absence of RF and ACPA constitutes a major barrier for patients with seronegative RA. Several cohorts, including ESPOIR and Leiden-EAC, have shown that a significant number of ACPA− RF− patients with typical inflammatory joint symptoms did not fulfill the 2010 ACR/EULAR criteria at diagnosis [30]. These findings raise concerns about the sensitivity of the criteria in seronegative patients, particularly at the early disease stage, with the risk of underdiagnosis or delayed treatment initiation.

### 5.2. Importance of the Erosive Profile

Some seronegative patients present with, or later develop, radiographic erosions characteristic of RA without initially being classified as such. This limitation arises from the fact that erosions are not included in the 2010 ACR/EULAR scoring system. To address this issue, an addendum to the ACR/EULAR criteria was published in 2013, stating that the presence of typical erosions on radiographs affecting at least three distinct joints among the proximal interphalangeal, metacarpophalangeal, metatarsophalangeal joints, or the wrist (counted as a single joint) is sufficient to classify a patient as having RA, even if the total score of 6 points is not met [30].

This evolution underscores the need for a more integrative diagnostic approach, combining clinical, biological, and imaging assessments, particularly for seronegative forms. Modern imaging techniques (MRI and ultrasound), which are more sensitive than conventional radiography for detecting early erosions [38,39,40,41,42,43,44], may play a central role in identifying erosive forms not captured by current classification criteria [38,39,40].

MRI studies have also highlighted important differences in the anatomical distribution of bone erosions according to serostatus. In a large cohort of early RA, Mo et al. demonstrated that seronegative patients exhibited preferential erosive involvement of the scaphoid and lunate, whereas seropositive patients more commonly showed erosions affecting the capitate and hamate. This distinct spatial pattern supports the existence of serology-associated structural phenotypes and illustrates the ability of MRI to detect joint damage distributions that remain invisible to conventional radiography. Such differences emphasize the contribution of imaging to refining structural characterization and identifying patients at higher risk of erosive progression across both seronegative and seropositive subsets [38,39,40,41,42,43,44,45,46,47].

### 5.3. Diagnostic Uncertainty and Serological Stability in Early Seronegative RA

In contrast to serology, musculoskeletal ultrasound (MSUS) offers a highly sensitive means of capturing the inflammatory and erosive phenotype of seronegative RA. High-frequency ultrasound studies consistently demonstrate that SNRA is clearly distinct from osteoarthritis, with markedly higher prevalence and severity of synovitis, power Doppler (PD) signal, tenosynovitis, and bone erosions. Importantly, ultrasound scores correlate with DAS28 and acute-phase reactants only in seronegative RA, underscoring its diagnostic relevance when serology is uninformative.

Cen et al. further showed that although inflammatory ultrasound features are broadly similar between seronegative and seropositive RA, bone erosions are more frequently detected in patients with high anti-CCP titres [46]. This suggests a continuum between seronegative and seropositive disease rather than two fully distinct entities [46,47].

These findings were formalised by Xu et al. using a semi-quantitative scoring system assessing synovial hypertrophy, PD signal, and erosions in a large cohort: global ultrasound scores did not differ significantly between seronegative and seropositive RA, but sharply distinguished SNRA from non-RA conditions. In serology-negative patients, the presence of at least one joint with PD grade ≥ 2 or erosion grade ≥ 2 achieved both high sensitivity and high specificity for RA [44,45].

Together, these data indicate that MSUS can effectively compensate for negative serology, differentiate seronegative RA from degenerative disease, and reveal an imaging phenotype that in many respects overlaps with that of seropositive RA.

Similarly, serological status (RF and ACPA) should not be regarded as a fixed or definitive attribute, but rather as a dynamic marker whose fluctuations remain uncommon. In the ESPOIR early-arthritis cohort, Gossec et al. reported only 2.6% anti-CCP seroconversion to positivity over two years among initially negative patients, and 4.6% IgM-RF seroconversion [48]. A Danish early-arthritis study confirmed comparably low rates, with approximately 2% seroconversion for ACPA and 5% for RF during follow-up [49].

In established RA, Hiwa et al. observed that only 5.4% of ACPA-negative patients became ACPA-positive over time, and that this occurred exclusively in RF-positive individuals; no seroconversions were detected among double-negative patients [50]. A prospective study evaluating the utility of repeating ACPA testing further demonstrated that persistent ACPA−→ACPA+ seroconversion after diagnosis is exceedingly rare—estimated at <1% in double-negative patients—and that routine repeat testing offers no clinical or cost-effective benefit in the absence of new clinical findings [51].

Taken together, these data support the view that serostatus functions as a probabilistic marker rather than a definitive diagnostic anchor. Seroconversion does occur but remains rare, particularly in double-negative patients, and does not justify systematic repeat RF or ACPA testing in routine clinical practice (Table 2).

### 5.4. Diagnostic Delay and Treatment Window in Seronegative RA

A consistent body of evidence indicates that seronegative rheumatoid arthritis (RA) is disproportionately affected by delays in both clinical diagnosis and initiation of disease-modifying antirheumatic drugs (DMARDs) [52,53,54,55]. In the Mayo Clinic cohort, Coffey et al. showed that seronegative patients experienced substantially longer delays before a confirmed clinical diagnosis of RA than their seropositive counterparts (median 187 vs. 11 days). A similar pattern was observed for treatment initiation: while seropositive patients typically commenced DMARD therapy within a few weeks of first joint swelling, seronegative patients waited considerably longer (median 40 vs. 14 days), underscoring a systematic lag in recognising and treating disease activity in the absence of autoantibodies [52].

These delays are clinically meaningful, as they extend beyond administrative timelines and directly compromise access to the therapeutic “window of opportunity,” during which early intervention is most likely to prevent long-term structural damage and optimised remission rates. In the absence of rheumatoid factor (RF) or anti-citrullinated peptide antibody (ACPA) positivity, seronegative RA is therefore at heightened risk of missing this critical window—not because the disease is inherently milder, but because inflammation is slower to be recognised and labelled as RA [52].

In conclusion, these limitations call for a re-evaluation of current classification criteria, particularly in an era in which seronegative forms appear to be increasing in relative prevalence.

## 6. Differential Diagnosis

The diagnosis of seronegative rheumatoid arthritis (SNRA) remains particularly challenging owing to the absence of specific serological biomarkers and the striking heterogeneity of its clinical presentations. As a result, numerous inflammatory, metabolic, infectious, and neoplastic conditions may closely mimic SNRA (Table 3). Accurate diagnosis, therefore, requires an iterative, multimodal approach that integrates detailed clinical evaluation, targeted laboratory testing, and advanced imaging modalities [52,53,54,55,56,57,58,59,60,61,62,63,64,65,66,67].

One of the most frequent and clinically relevant mimics is calcium pyrophosphate deposition disease (CPPD), especially in its polyarticular form. Krekeler et al. demonstrated that radiographic CPPD deposits were significantly more common in patients initially labelled as SNRA than in seropositive RA, with more than one-quarter ultimately meeting criteria for CPPD [54]. This misclassification often stemmed from the absence of synovial fluid analysis, underscoring the importance of joint aspiration in suspected SNRA when crystal disease is a possibility [53,54].

Spondyloarthritides—particularly psoriatic arthritis (PsA)—represent another major diagnostic pitfall. PsA may present as symmetric polyarthritis mimicking RA, especially early in the disease and in the absence of cutaneous psoriasis. Clues favouring PsA or other spondyloarthritides include asymmetric involvement, enthesitis, dactylitis, predominant lower-limb disease, nail changes, inflammatory axial pain, or HLA-B27 positivity. MRI can help distinguish PsA from RA by revealing peri-tendon inflammation, distal joint involvement, or bone proliferation patterns that are atypical for SNRA [55].

Infectious arthritides must also be considered. Viral infections such as parvovirus B19, chikungunya, or hepatitis B and C can induce transient inflammatory polyarthritis clinically indistinguishable from early SNRA. Recognition of systemic features, epidemiological context, and spontaneous resolution can avoid misdiagnosis and inappropriate immunosuppression [56].

Whipple’s disease deserves particular attention despite its rarity. *Tropheryma whipplei* infection can manifest as chronic, seronegative, non-erosive polyarthritis that responds poorly to immunosuppression. The presence of chronic diarrhea, weight loss, fever, or migratory oligoarthritis should prompt consideration of this entity. PCR testing—preferably from duodenal or synovial tissue—remains the diagnostic gold standard, and targeted antibiotic therapy is required for disease control [57].

In patients with antinuclear antibodies (ANA) and features such as Raynaud’s phenomenon, rashes, or interstitial lung involvement, underlying connective tissue diseases (CTDs) such as systemic lupus erythematosus, Sjögren’s disease, or systemic sclerosis must be excluded. These conditions may initially present with inflammatory arthritis and evolve over time, necessitating longitudinal surveillance [56].

Polymyalgia rheumatica (PMR) is another important mimicker, particularly when presenting with polyarticular pain and stiffness. The predominant involvement of the shoulder and pelvic girdles, elevated inflammatory markers, and rapid response to low-dose glucocorticoids typically help distinguish PMR from RA [58].

Neoplastic diseases also warrant consideration. Lymphoproliferative disorders and solid tumours can present with inflammatory arthritis or seronegative polyarthritis, sometimes preceding cancer diagnosis. Conversely, cancer therapies—most notably immune checkpoint inhibitors—can induce RA-like inflammatory arthritis that often lacks autoantibodies and may persist after treatment discontinuation. Paraneoplastic arthritis may remit following effective tumour management, offering an important diagnostic clue [59,60,61,62,63,64,65,66].

Differentiation from erosive osteoarthritis (EOA) can also be challenging. EOA is characterized by mechanical pain, absence of systemic inflammation, and central “gull-wing” erosions—features that contrast with the marginal erosions typical of RA. DIP involvement is common in EOA but unusual in RA, and carpal erosions are rare [67].

Long-term data highlight the consequences of diagnostic uncertainty. In a 10-year follow-up of 435 patients initially classified as seronegative RA, only 2.9% were ultimately confirmed to have true RA. Most were eventually reclassified as PMR, giant cell arteritis, spondyloarthritis, PsA, crystal arthropathy, EOA, paraneoplastic disease, or undifferentiated arthritis. This high reclassification rate illustrates the need for careful, dynamic diagnostic reassessment over time [67].

**Figure 2:**
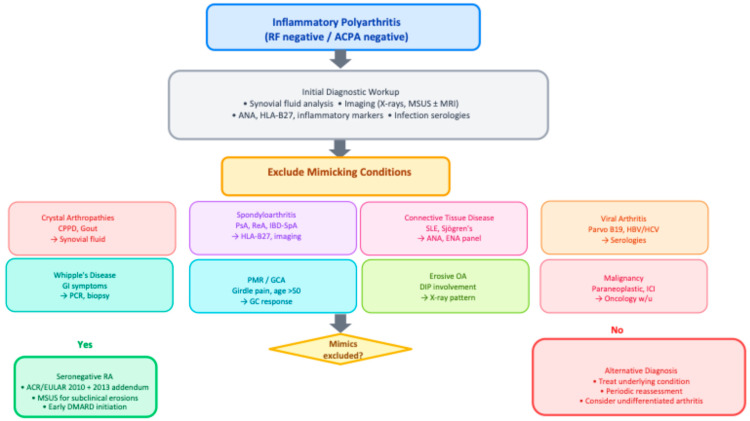
Diagnostic algorithm for seronegative inflammatory polyarthritis. Abbreviations: ACPA, anti-citrullinated peptide antibodies; CPPD, calcium pyrophosphate deposition disease; CTD, connective tissue disease; DIP, distal interphalangeal; GC, glucocorticoid; GCA, giant cell arteritis; HBV/HCV, hepatitis B/C virus; IBD-SpA, inflammatory bowel disease-associated spondyloarthritis; ICI, immune checkpoint inhibitor; MSUS, musculoskeletal ultrasound; OA, osteoarthritis; PMR, polymyalgia rheumatica; PsA, psoriatic arthritis; ReA, reactive arthritis; RF, rheumatoid factor; SLE, systemic lupus erythematosus.

In summary, seronegative polyarthritis encompasses a broad array of mimicking conditions, many of which are more common than true SNRA. Accurate diagnosis requires a cautious and iterative process, with regular re-evaluation based on clinical evolution, treatment response, and imaging. Particular vigilance is needed for oncology-related mimics—both paraneoplastic arthritis and immune checkpoint inhibitor–induced arthritis—which may be clinically identical to SNRA at presentation. A comprehensive differential diagnosis must therefore be systematically explored and excluded depending on the clinical context, as summarized in Figure 2.

## 7. Bone Erosions in Rheumatoid Arthritis

### 7.1. Pathophysiology and Clinical Significance

RA is characterized by the presence of bone erosions, a central marker of functional prognosis. These erosions represent localized bone loss (osteolysis) resulting from an imbalance between osteoclast-mediated resorption and osteoblast-driven bone formation. This process occurs in the context of both systemic and localized inflammation, where synovitis produces excess proinflammatory cytokines and RANKL, stimulating osteoclast differentiation [68,69].

This interaction between inflammation and bone resorption is central to the concept of osteoimmunology, which establishes a direct link between chronic immune activation and deleterious bone remodeling. Cytokines such as TNF-α, IL-1, IL-6, IL-17, and IL-15 play major proerosive roles by either directly activating osteoclast precursors or stimulating mesenchymal cells to produce RANKL. Conversely, cytokines such as IL-33, IL-12, and interferons (IFN-γ, type I IFNs) exert inhibitory effects on bone resorption, but these remain insufficient to counterbalance the inflammatory milieu of active RA [70].

Beyond inflammation, experimental data suggests that autoantibodies, particularly ACPA, have a direct role in osteoclastogenesis [69,70,71,72,73]. By binding to citrullinated proteins on the surface of osteoclast precursors, ACPA stimulates their differentiation independently of systemic inflammation. This mechanism is further enhanced by macrophage activation through immune complexes, leading to increased TNF and RANKL production. This link between ACPA and structural damage positions these antibodies as prognostic biomarkers beyond their diagnostic value [71,72,73].

### 7.2. Localization and Pattern of Erosions

**Figure 3:**
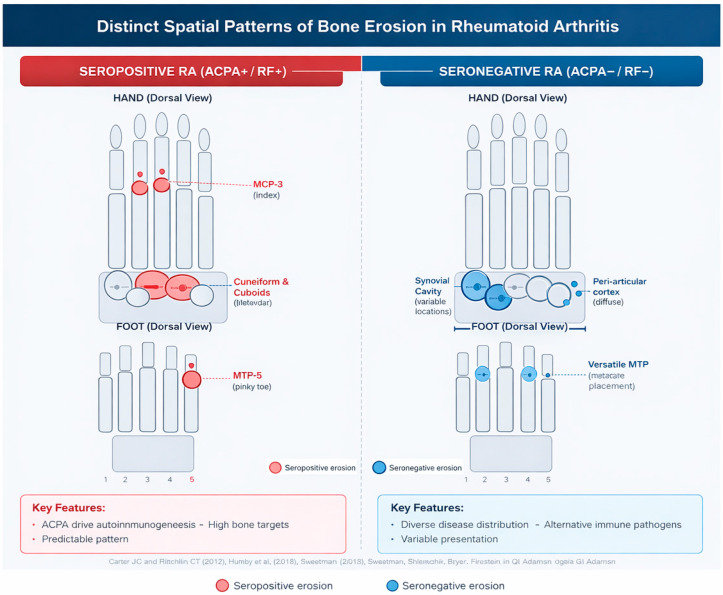
Seropositive rheumatoid arthritis (ACPA+/RF+) shows a characteristic erosive pattern involving the capitate, hamate, the 2nd and 3rd metacarpophalangeal (MCP) joints, and the 5th metatarsophalangeal (MTP5) joint. In contrast, seronegative rheumatoid arthritis (ACPA−/RF−) exhibits a more heterogeneous distribution, with erosions more frequently observed in the scaphoid and lunate, variable involvement of the lesser MTP joints, and occasional carpal shortening. These distinct spatial patterns reflect the different immunopathological mechanisms driving structural damage in seropositive versus seronegative disease refs. [74,75,76].

Several studies have explored the extent to which autoantibody status shapes the structural damage pattern in rheumatoid arthritis (RA). In a multicentre cohort comparing ACPA-positive and ACPA-negative patients, Grosse et al. reported a markedly higher erosive burden in ACPA-positive disease, with modified Sharp erosion scores on radiography being approximately four-fold higher and total ultrasound erosion scores more than four-fold higher than in ACPA-negative RA. Bilateral erosions of the fifth metatarsophalangeal joint (MTP5) were particularly discriminant, being present in almost all ACPA-positive patients on both radiographs and ultrasound, and ACPA positivity remained independently associated with erosive RA in multivariable analyses. Complementary data from Gadeholt et al. showed that seropositive RA displays higher global erosion and joint space narrowing scores than seronegative RA, with preferential involvement of the feet and metacarpophalangeal joints, whereas seronegative RA tends to show periarticular ossifications, carpal shortening and relative sparing of carpometacarpal joints. Together, these findings support the view that ACPA-positive RA represents a more strongly erosive entity at the group level and exhibits a distinct radiographic phenotype, while also highlighting the existence of ACPA-negative patients with significant structural damage whose characteristics remain to be fully delineated [74,75,76] (Figure 3).

#### Clinical Implications

These distinct erosion patterns support the concept of different immunopathological pathways underlying structural damage in seropositive and seronegative disease. Recognition of these patterns aids in diagnostic refinement, prognostic assessment, and may inform targeted therapeutic strategies tailored to the specific disease subtype (Table 4).

### 7.3. Prevalence and Progression According to Serological Status

In a post hoc analysis of the BARFOT cohort, which followed 608 patients with early RA for eight years, 24% never developed radiographic erosions, thus defining a non-erosive subgroup. This study also demonstrated a clear association between autoantibody status and structural damage: anti-CCP positivity was markedly more frequent in patients who became erosive than in those who remained erosion-free, and absence of anti-CCP emerged as an independent predictor of a non-erosive course [5]. However, in the absence of a detailed description of the overlap between RF and ACPA (double-negative patients versus patients negative for only one of the two markers), these data do not allow a precise estimation of the proportion of strictly seronegative patients (RF−/ACPA−) who remain non-erosive in the long term.

In the French ESPOIR cohort, the gradient of structural risk according to ACPA status was already apparent at baseline: 18.9% of ACPA− patients had erosions at inclusion versus 31.2% of ACPA+ patients. After three years, these proportions increased to 29% and 63%, respectively, confirming that ACPA positivity is associated with a substantially higher erosive risk, even though strictly seronegative patients are far from completely protected [22,26]. Similarly, in a cohort of 271 RA patients, Liao et al. reported that 21% remained erosion-free after more than two years of follow-up; in the same study, erosions nonetheless occurred in both seronegative and seropositive patients, underlining that the absence of autoantibodies does not preclude unfavourable structural progression [76].

The importance of early structural status is supported by French early RA data showing that radiographic progression during the first year is one of the strongest predictors of subsequent damage, outperforming autoantibody status, DAS28 and CRP. Patients without radiographic progression in the first year have a markedly lower long-term risk of erosion accrual [77].

Collectively, these observations highlight that while the presence of autoantibodies (particularly ACPA) is strongly associated with structural progression, their absence does not rule out destructive evolution. SNRA cannot, therefore, be considered benign by default: up to 30% of seronegative patients become erosive within five years, and a substantial proportion exhibit sufficient osteoclastic activity to induce early structural damage, sometimes independently of clinically perceived disease intensity. Under adequate management, however, strictly seronegative patients appear to have a lower average risk of structural progression than their seropositive counterparts [78].

## 8. Treatment Response

The therapeutic management of seronegative rheumatoid arthritis (SNRA) remains challenging. It relies predominantly on evidence derived from seropositive populations, making extrapolations uncertain (Table 5). Nevertheless, the treat-to-target strategy, based on regular assessment of disease activity, remains relevant for both seropositive and seronegative disease forms [79]. It is critical to acknowledge that most therapeutic trials in RA have been enriched for seropositive patients, and seronegative subgroups are often examined only in retrospective post hoc analyses with limited statistical power [80]. Furthermore, no trials have directly compared therapeutic strategies in erosive versus non-erosive SNRA. The treatment recommendations presented below should therefore be interpreted with appropriate caution and framed as hypothesis-generating rather than definitive guidance. Future trials specifically designed for seronegative populations, stratified by erosive status and AMPA profile, are urgently needed.

Available data suggest that early diagnosis and early initiation of therapy improve the prognosis of seronegative RA (SNRA), even though the therapeutic window of opportunity appears to be wider than in ACPA-positive patients [79]. Paradoxically, this inherently broader window is more frequently missed in routine practice due to the diagnostic and therapeutic delays already well documented in SNRA. Recently, a Perspective published in *Nature Reviews Rheumatology* further emphasized that seronegative RA remains poorly understood and under-recognized, with diagnostic delays and unmet needs that justify renewed attention as well as therapeutic strategies specifically tailored to this subgroup [80].

### 8.1. Glucocorticoids

The role of glucocorticoids remains debated. Their main advantage lies in the rapid control of symptoms and early prevention of structural progression [80]. The GLORIA study demonstrated their efficacy and favorable safety profile, even in elderly patients [81].

### 8.2. Response to csDMARDs

Conventional synthetic DMARDs (csDMARDs), such as methotrexate (MTX), leflunomide, sulfasalazine, and hydroxychloroquine, are recommended regardless of serological status. Several cohort studies, including ESPOIR, have shown similar EULAR response rates between SNRA and SPRA, although potential biases exist (treatment strategies, classification criteria) [82].

Methotrexate is generally considered the anchor drug, but its efficacy may be slightly lower in SNRA. Analysis of data from four randomized trials by Duong et al. suggested that ACPA positivity is a predictor of better response to MTX. Moreover, seronegative patients may exhibit a slower response [83].

### 8.3. Response to bDMARDs and tsDMARDs

Biological DMARDs (bDMARDs) and targeted synthetic DMARDs (tsDMARDs) have transformed RA management, but their efficacy may vary by serological status due to the different roles of adaptive versus innate immunity in SPRA and SNRA.

#### 8.3.1. Abatacept

Several studies have shown greater efficacy of abatacept in ACPA-positive patients. This benefit has been observed in both short-term prospective trials and long-term registry data. ACPA titers appear positively correlated with clinical response [84,85]. After one year of abatacept therapy, however, disease erosiveness does not appear to be significantly influenced by ACPA status [86].

#### 8.3.2. Rituximab

ACPA-positive patients treated with rituximab achieve better clinical responses and higher drug retention rates compared with ACPA-negative patients. Nonetheless, these differences become less pronounced beyond three years of follow-up [2,84,85].

#### 8.3.3. Tofacitinib and Other JAK Inhibitors

Data on JAK inhibitors remain limited. Retrospective analyses suggest higher retention rates of tofacitinib in ACPA-positive patients, although efficacy measured by disease activity scores or joint counts does not show significant differences [87]. A post hoc analysis of five randomized trials demonstrated that tofacitinib is effective regardless of serological status, though seropositive patients (ACPA+/RF+) achieved slightly higher response rates [87,88]. Limited data are available for other JAK inhibitors, although they represent promising therapeutic options.

#### 8.3.4. Anti-TNFα and Anti-IL-6 Agents

Unlike abatacept or rituximab, anti-TNFα and anti-IL-6R therapies appear to demonstrate efficacy largely independent of serological status [2,83,84,86,87,88,89,90]. Furthermore, analysis by Shipa et al. suggested that switching from one anti-TNF to another may be more effective in seronegative than in seropositive patients [89]. Preclinical data on sarilumab have also shown significant protective effects on joint structures, reducing synovitis, pannus formation, and bone erosions [90].

### 8.4. Effects of RA Therapies on Autoantibody Generation

An important and underexplored question is whether RA therapies themselves influence the generation, persistence, or titre of autoantibodies. Several lines of evidence suggest that immunomodulatory treatments can modulate humoral autoimmunity:

Rituximab, by depleting CD20+ B cells, has been shown to reduce ACPA and RF titres in a proportion of treated patients, though complete seroreversion is uncommon. The degree of autoantibody reduction correlates variably with clinical response, and some patients achieve clinical remission despite persistent autoantibody positivity [85,87].

Abatacept, through inhibition of T-cell co-stimulation, may indirectly affect B-cell function and autoantibody production. Studies have reported modest reductions in ACPA titres with sustained abatacept therapy, potentially reflecting disruption of T-cell help for autoreactive B cells [86].

TNF inhibitors and IL-6 receptor antagonists do not appear to consistently modify autoantibody titres, though individual variation exists. JAK inhibitors may theoretically affect B-cell signalling and immunoglobulin production, but data on their effects on ACPA and RF remain limited [87,89].

Methotrexate and other conventional DMARDs have variable reported effects on autoantibody levels, with some studies suggesting gradual titre reduction during sustained remission [82,83].

The clinical implications of treatment-induced autoantibody modulation remain unclear. Whether achieving ACPA or RF negativity during therapy (‘seroreversion’) confers additional prognostic benefit beyond clinical remission is an area of active investigation. For seronegative patients, the effects of therapy on non-conventional AMPA (anti-CarP, anti-acetylated proteins) have not been systematically studied but represent an important research priority.

### 8.5. Recommendations for Erosive Seronegative Forms

To date, no study has directly compared therapeutic responses between erosive and non-erosive seronegative RA (SNRA), leaving a critical evidence gap. The proposed algorithms for erosive versus non-erosive SNRA should therefore be understood as expert-informed frameworks based on indirect evidence and pathophysiological reasoning, rather than recommendations derived from high-quality comparative trials. Nevertheless, the consistently higher risk of structural progression in erosive SNRA supports a more proactive management strategy. Early initiation of csDMARDs—ideally within the first three months of symptom onset—remains essential, as delays disproportionately affect seronegative patients who are already at risk of diagnostic lag. Extended AMPA profiling may eventually contribute to treatment stratification within SNRA. Patients who are RF/ACPA-negative but positive for anti-CarP or other AMPA may warrant earlier escalation to bDMARDs or tsDMARDs, analogous to seropositive disease. Conversely, truly autoantibody-negative patients with non-erosive phenotypes may be appropriate candidates for less aggressive approaches with close monitoring. These hypotheses require prospective validation.

**Figure 4:**
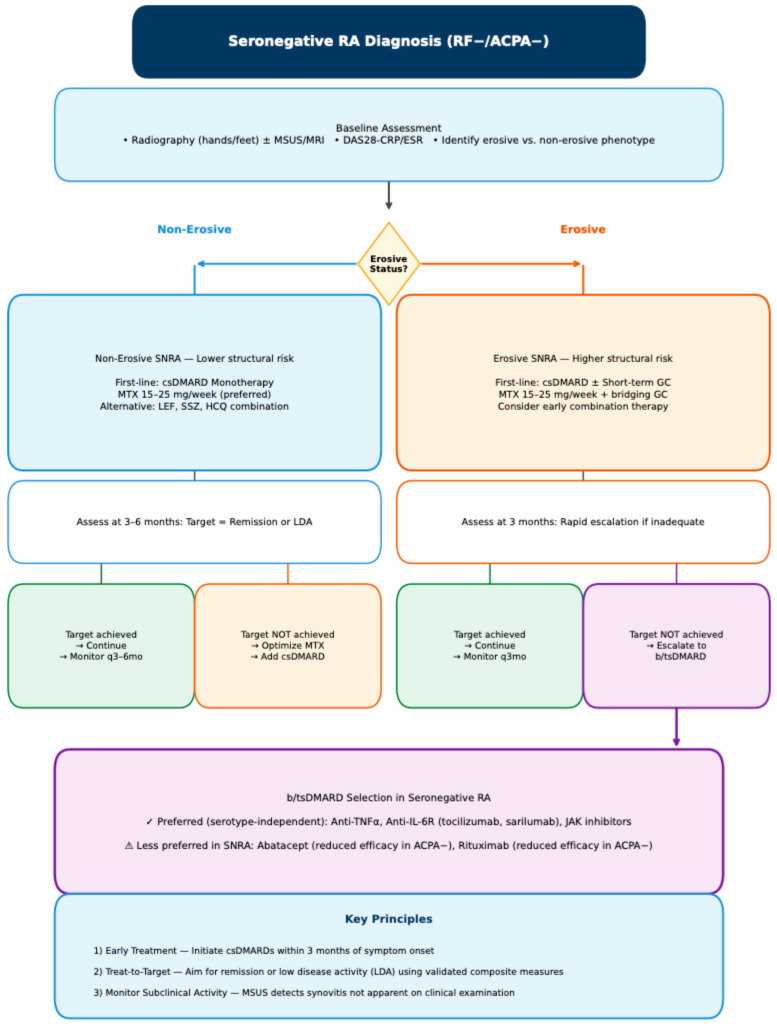
Proposed diagnostic algorithm for treatment in Seronegative RA. Abbreviations: ACPA, anti-citrullinated peptide antibodies; b/tsDMARD, biologic/targeted synthetic DMARD; csDMARD, conventional synthetic DMARD; DAS28, Disease Activity Score 28; GC, glucocorticoid; HCQ, hydroxychloroquine; IL-6R, interleukin-6 receptor; JAK, Janus kinase; LDA, low disease activity; LEF, leflunomide; MSUS, musculoskeletal ultrasound; MTX, methotrexate; RF, rheumatoid factor; SNRA, seronegative rheumatoid arthritis; SSZ, sulfasalazine; TNF, tumor necrosis factor.

Close monitoring of clinical activity and serial imaging (ultrasound or MRI) is crucial, and early escalation to bDMARDs or tsDMARDs should be considered promptly when structural progression or insufficient disease control becomes apparent. Effective suppression of synovitis is central to preventing erosive damage. Importantly, imaging-detected subclinical inflammation may persist despite apparent clinical remission, particularly in seronegative disease. Such residual synovitis has been shown to correlate with ongoing osteoclast activation and should therefore influence therapeutic decisions, even in patients who meet clinical remission criteria. Cytokine inhibition—especially targeting TNF and IL-6—as well as small-molecule blockade of intracellular signalling pathways such as JAK, SYK, and BTK, has demonstrated significant protective effects against structural damage. These agents reduce osteoclast differentiation both indirectly through inflammation control and directly through modulation of bone-resorptive pathways [87,88,89,90,91,92,93]. This dual mechanism provides a strong rationale for their use in erosive SNRA, where the threshold for treatment intensification should arguably be lower than in non-erosive forms (Figure 4).

## 9. Conclusions

Seronegative rheumatoid arthritis (SNRA) remains a diagnostically and prognostically challenging entity. Long regarded as a milder form due to the absence of autoantibodies, it encompasses in fact substantial heterogeneity, with highly variable disease trajectories. While some non-erosive forms carry a favorable functional and structural prognosis, erosive subsets may progress to severe joint destruction comparable to that seen in seropositive patients. This diversity complicates therapeutic management and highlights the need for improved stratification of patients according to their risk of progression.

From a therapeutic perspective, available evidence suggests that B- or T-cell-targeted agents, such as rituximab or abatacept, are generally less effective in seronegative patients, whereas cytokine inhibitors (anti-TNFα, anti-IL-6) and JAK inhibitors appear to offer more consistent efficacy, irrespective of serological status. However, most studies do not differentiate treatment responses according to the presence or absence of erosions, despite their value as an important marker of disease severity.

In erosive forms, the issue of early treatment intensification is particularly pressing. Inflammatory control and the rapid initiation of csDMARD therapy, ideally within three months of symptom onset, remain a priority. In cases of insufficient response, prompt escalation to bDMARDs or tsDMARDs should be considered. Yet, current guidelines do not incorporate the erosive/non-erosive distinction into therapeutic algorithms, owing to the lack of specific evidence. Furthermore, the 2010 ACR/EULAR criteria are not fully suited to seronegative forms, given the strong weighting of serology, which may delay both diagnosis and timely management in patients with progressive disease.

It is therefore imperative to better characterize therapeutic response profiles in SNRA patients, accounting not only for serological status but also for the presence or absence of erosions at diagnosis. Distinguishing between erosive and non-erosive SNRA could become a decisive tool to guide therapeutic decision-making, optimize treatment strategies, and advance personalized medicine.

## 10. Perspectives and Future Directions

The evolving understanding of seronegative rheumatoid arthritis opens several avenues for future research and clinical practice improvements.

First, rigorous prospective studies or registry-based analyses specifically addressing treatment responses according to erosive status in SNRA would fill an important gap in the current literature and could have major clinical implications. Such studies should stratify patients by baseline erosive status and track both clinical and structural outcomes over extended follow-up periods.

Second, the development of novel biomarkers capable of predicting erosive risk in seronegative patients represents a pressing unmet need. Beyond anti-CarP and other non-conventional autoantibodies, multi-omic approaches integrating transcriptomic, proteomic, and metabolomic signatures may enable more precise risk stratification and earlier identification of patients requiring aggressive therapy.

Third, the distinct immunopathological profile of SNRA—characterized by innate immune predominance—suggests potential therapeutic targets beyond current T- and B-cell directed approaches. Agents targeting macrophage activation, dendritic cell function, or fibroblast-driven pathways merit exploration in this population.

Fourth, revision of the ACR/EULAR classification criteria to better accommodate seronegative presentations warrants consideration. Integration of advanced imaging findings, particularly ultrasound-detected erosions and synovitis, could improve diagnostic sensitivity and reduce the delays that currently compromise outcomes in this subset.

Finally, real-world data from national registries should systematically capture serological status and erosive phenotype to enable large-scale comparative effectiveness research across the spectrum of RA presentations.

## 11. Limitations

This review has several important limitations that should be acknowledged. First, the narrative design inherently restricts methodological rigor: study selection was not performed according to PRISMA standards, and neither risk-of-bias assessment nor formal evaluation of publication bias was possible. As a result, the strength of evidence supporting certain conclusions may be influenced by selective publication or the overrepresentation of positive findings.

Second, substantial heterogeneity exists in the definition of “seronegative RA” across the literature. Some cohorts classify patients solely by RF negativity, others by double-negativity for RF and ACPA, while several older studies lack high-sensitivity second- or third-generation assays. This variability creates conceptual noise, complicates cross-study comparison, and may obscure true biological distinctions within the seronegative spectrum. The absence of uniform serological definitions likely inflates heterogeneity in clinical outcomes and limits the validity of pooled interpretations.

Third, and critically, the conventional RF/ACPA-based definition of seronegativity does not capture the full spectrum of humoral autoimmunity in RA. Extended AMPA profiling—including anti-CarP, anti-acetylated protein, and anti-MAA antibodies—was not routinely available in most historical cohorts included in this review. Consequently, an unknown proportion of patients classified as ‘seronegative’ may harbour unconventional autoantibodies that were simply not measured. This limitation fundamentally affects the interpretation of clinical, imaging, and therapeutic outcomes attributed to ‘seronegative’ disease. The equation of RF/ACPA negativity with true immunonegativity is biologically inaccurate, and readers should interpret conclusions about SNRA with this caveat in mind.

Fourth, qualitative features of autoantibody responses—including isotype distribution (IgA versus IgG, IgG subclasses) and fine specificity profiles—were not systematically addressed in the cohorts reviewed. These dimensions represent additional layers of immunological heterogeneity that may influence prognosis and treatment response but remain underexplored in seronegative RA.

Fifth, most therapeutic trials in RA have historically been enriched in seropositive patients, reflecting recruitment strategies that favored classical biomarker-positive disease. Seronegative patients were often included only as small subgroups or examined in retrospective post hoc analyses. This markedly reduces statistical power, introduces survivor and responder bias, and limits generalizability of therapeutic conclusions to the true seronegative population—particularly to erosive subsets, which are rarely examined independently.

Sixth, secular trends in the classification of early arthritis—including the transition from the 1987 to the 2010 ACR/EULAR criteria—introduce significant historical bias. Changes in autoantibody assay sensitivity and imaging practice (especially ultrasound and MRI) further complicate comparisons between older and more contemporary cohorts. Consequently, observed differences in disease trajectory may reflect methodological evolution as much as biological reality.

Seventh, most available evidence derives from European and North American cohorts, where genetic, environmental, and lifestyle determinants differ substantially from those in Asian, African, or South American populations. As seronegative RA appears particularly influenced by environmental factors, the external validity of current findings may be limited, and global prevalence or phenotype distributions remain insufficiently characterized.

Finally, and perhaps most critically, reliable biomarkers for predicting erosive evolution in seronegative RA remain lacking. No validated imaging, serological, or molecular signature currently allows stratification of erosive vs. non-erosive trajectories. The integration of AMPA profiling, autoantibody isotype analysis, and multi-omic approaches represents a promising direction but has not yet been validated for clinical use in seronegative populations. This gap limits the development of personalized treatment strategies and undermines the design of targeted clinical trials focused specifically on seronegative disease. The present review highlights this unmet need but cannot resolve it given the current state of the literature.

**KEY MESSAGES**

Seronegative RA is not uniformly benign: up to 30% of patients develop erosive disease within 5 years, with structural outcomes that may rival seropositive RA.

- RF/ACPA negativity does not equate to true immunonegativity: the AMPA framework demonstrates that a substantial proportion of conventionally defined ‘seronegative’ patients harbour autoantibodies against carbamylated, acetylated, and other modified proteins not captured by standard testing.
- Infectious triggers, particularly periodontal pathogens, may contribute to RA pathogenesis through distinct citrullination pathways and systemic translocation of bacterial virulence factors.
- Diagnostic delays are systematic: seronegative patients experience longer time to diagnosis (median 187 vs. 11 days) and treatment initiation, risking the therapeutic window of opportunity.
- Imaging is essential: musculoskeletal ultrasound and MRI effectively compensate for negative serology and should be incorporated into diagnostic algorithms.
- Treatment selection matters: anti-TNF, anti-IL-6, and JAK inhibitors show consistent efficacy regardless of serostatus, whereas rituximab and abatacept are less effective in seronegative patients.
- RA therapies may modulate autoantibody generation, though the clinical implications of treatment-induced serological changes remain to be fully characterized.
- Therapeutic recommendations for erosive vs. non-erosive SNRA are hypothesis-generating: no trials have directly compared strategies in these subgroups, and proposed algorithms should be interpreted with appropriate caution.

## 12. Methods

This narrative review included full-text original articles obtained through PubMed, Cochrane, and Google Scholar. Specific keywords were used to identify eligible studies: Rheumatoid arthritis, Seronegative, Autoantibody-negative, Seropositive, Autoantibody-positive, Erosive, Erosion. The studies were limited to randomized controlled trials and literature reviews published mainly over the past 10 years, in English, and focusing on human subjects. Case reports, case series, and conference abstracts were excluded.

## Figures and Tables

**Table 1:** Key Distinctions Between Seropositive (SPRA) and Seronegative Rheumatoid Arthritis (SNRA).

Dimension	Seropositive RA (RF+/ACPA+)	Seronegative RA (RF−/ACPA−)
Dominant immunopathology	Adaptive immune activation (T–B interaction, TLS).	Innate immune–dominant synovitis; absence of TLS.
Autoantibody profile	ACPA, RF, anti-PAD4; broad AMPA signature.	Anti-CarP (7–29%), occasional anti-RA33; limited humoral autoimmunity.
Genetic susceptibility	Strong HLA-DRB1 shared epitope.	Weak HLA influence; stronger environmental modulation.
Synovial phenotype	Lymphoplasmacytic, B-cell–rich aggregates.	Macrophage-rich, dendritic-cell–driven; fibroblast activation.
Structural severity	High erosive risk (~63% at 3 years).	Moderate erosive risk (~29% at 3 years).
Erosion pattern	Capitate, hamate, MCP joints.	Scaphoid, lunate, MTP; heterogeneous distribution.
Extra-articular features	ILD, nodules, vasculitis frequent.	ILD rare; fewer extra-articular manifestations.
2010 ACR/EULAR criteria	Serology drives rapid classification.	Often under-classified; requires higher joint counts.
Therapeutic response	Best responses to abatacept and rituximab.	Better responses to TNFi and IL-6 inhibitors.
Window of opportunity	Usually captured early.	Often missed due to diagnostic delay.
Overall prognosis	Structurally aggressive trajectory.	Heterogeneous course; 20–30% become erosive.

ACPA = anti-citrullinated peptide antibodies; anti-CarP = anti-carbamylated protein antibodies; anti-PAD4 = anti-peptidyl arginine deiminase 4 antibodies; ILD = interstitial lung disease.

**Table 2:** Distinguishing Erosive vs. Non-Erosive Seronegative RA (SNRA).

Dimension	EROSIVE SNRA	NON-EROSIVE SNRA
Initial clinical phenotype	Persistent synovitis; higher DAS28; prolonged morning stiffness	Moderate inflammatory activity; fluctuating symptoms
Symptom duration before therapy	Longer (>12 weeks); diagnosis often delayed	Shorter, earlier recognition
Major predictors	High CRP/ESR; male sex; smoking; baseline erosions	Low-grade inflammation; rapid early response
Ultrasound features	Power Doppler ≥ 2; erosions grade ≥ 2; multi-joint involvement	Minimal Doppler signal; no significant erosions
MRI characteristics	Bone marrow oedema + synovitis + early erosions	Oedema minimal/absent; structurally stable
Non-conventional autoantibodies	Anti-CarP sometimes positive; broader PTM signature	Typically negative
Synovial biology	Activated macrophages, aggressive fibroblast subsets	Mild hyperplasia; lower inflammatory load
Radiographic progression	High (20–30% at 2–3 years)	Minimal; long-term stability
Treatment response	Often requires early intensification: MTX → bDMARD/tsDMARD	Good MTX response; few switches
Optimal strategy	Tight control, early escalation, imaging-guided monitoring	Treat-to-target with broader intervals
Long-term prognosis	Higher risk of structural destruction & disability	Excellent long-term outcomes

**Table 3:** Differential Diagnosis of Seronegative Polyarthritis.

CONDITION	KEY DISTINGUISHING FEATURES	DIAGNOSTIC APPROACH
CPPD	Chondrocalcinosis (wrists, MCPs, knees); pseudo-RA; common in elderly; up to 25% of SNRA may be CPPD masquerades.	Hand/wrist X-rays; ultrasound for CPP deposits; synovial fluid crystal analysis.
Psoriatic arthritis	Asymmetric arthritis, DIP involvement, dactylitis, enthesitis; nail pitting; psoriasis may be absent (“hidden psoriasis”).	Full skin + nail exam; family history; HLA-B27; MRI for enthesitis/sacroiliitis.
Axial SpA/IBD-SpA	Lower-limb arthritis; inflammatory back pain; heel enthesitis; uveitis; GI symptoms (diarrhea, abdominal pain).	HLA-B27; SI-joint MRI; colonoscopy if GI symptoms.
Viral arthritis	Acute, symmetric, transient arthritis; parvovirus B19, chikungunya, HBV/HCV, HIV.	Viral serologies; epidemiological context; follow-up to confirm spontaneous resolution.
Whipple disease	Chronic seronegative non-erosive polyarthritis; weight loss; diarrhea; paradoxical worsening with steroids.	Duodenal or synovial PCR for *T. whipplei*.
Connective tissue diseases	ANA positivity; Raynaud; sicca; rashes; ILD; episodic inflammatory arthritis.	ANA + ENA panel; capillaroscopy; HRCT if pulmonary signs.
Polymyalgia rheumatica	Rhizomelic pain/stiffness (shoulders/pelvis); age > 50; rapid response to low-dose glucocorticoids.	ESR/CRP; trial of low-dose GC; assess for GCA.

**Table 4:** Distinctive radiographic patterns in seropositive and seronegative rheumatoid arthritis. This table summarizes the distinct spatial patterns of bone erosions according to serological status as documented by Grosse et al. [74], Gadeholt et al. [75], and Liao et al. [76]. Seropositive RA (ACPA+/RF+) shows characteristic erosive involvement of the capitate, hamate, 2nd and 3rd metacarpophalangeal (MCP) joints, and bilateral 5th metatarsophalangeal (MTP5) joints. Seronegative RA (ACPA−/RF−) exhibits more heterogeneous distribution with preferential scaphoid and lunate involvement, variable MTP erosions, and occasional peri-articular ossifications. These patterns reflect different immunopathological mechanisms driving structural damage. MCP = metacarpophalangeal; MTP = metatarsophalangeal.

Seropositive RA	Seronegative RA
Capitate & Hamate involvement	Scaphoid & Lunate involvement
MCP 2–3 erosions	Variable MTP erosions
Bilateral MTP 5 erosions	Peri-articular ossifications
Predictable pattern	Heterogeneous distribution

**Table 5:** Treatment Response by Serological Status and Mechanism of Action.

Drug Class	Seropositive RA (SPRA)	Seronegative RA (SNRA)	Clinical Implications
Methotrexate	Strong, well-established efficacy; seropositivity often associates with higher baseline disease activity.	Generally similar clinical response; some cohorts show slightly less radiographic progression overall.	First-line in both; in SNRA, monitor early for suboptimal response and consider timely treatment escalation.
Abatacept	Higher clinical efficacy and drug persistence, particularly in RF/ACPA-positive patients.	Reduced effectiveness and shorter drug survival compared with SPRA.	Preferential option in SPRA; in SNRA, alternative bDMARDs/tsDMARDs may be more suitable unless specific reasons.
Rituximab	Superior response and retention, especially in ACPA-positive RA.	Effective but with lower response rates and more non-responders.	Less attractive in early lines for SNRA; best reserved for later sequences once other mechanisms have been tried.
Anti-TNF agents	Robust efficacy across serotypes; large datasets report broadly similar outcomes.	Comparable clinical responses; TNFi are frequently used as first biologic in SNRA.	Reasonable first bDMARD regardless of serostatus; TNFi-to-TNFi cycling may be useful in SNRA before class switch.
An-ti-IL-6R	Highly effective, including in difficult-to-treat SPRA; strong evidence for structural and bone protection.	Similar clinical efficacy; radiographic protection appears preserved.	Excellent option in SNRA; suitable when TNFi or B-cell–targeted therapies underperform.
JAK inhibitors	Effective with high response and retention; some cohorts show slightly better persistence in SPRA.	Effective with comparable clinical outcomes; some data suggest slightly lower drug survival.	Strong tsDMARD choice in SNRA, particularly after csDMARD and TNFi failure; retention and safety require follow-up.

ACPA = anti-citrullinated peptide antibodies; bDMARD = biological disease-modifying antirheumatic drug; IL-6R = interleukin-6 receptor; JAK = Janus kinase; SNRA = seronegative rheumatoid arthritis; TNF = tumor necrosis factor; TNFi = TNF inhibitor; tsDMARD = targeted synthetic disease-modifying antirheumatic drug.

## Data Availability

No new data were created or analyzed in this study.

## References

[1] Finckh A., Gilbert B., Hodkinson B., Bae S.C., Thomas R., Deane K.D., Alpizar-Rodriguez D., Lauper K. (2022). Global epidemiology of rheumatoid arthritis. Nat. Rev. Rheumatol..

[2] Courvoisier D.S., Chatzidionysiou K., Mongin D., Lauper K., Mariette X., Morel J., Gottenberg J.E., Pavelka K., Hyrich K.L., Strangfeld A. (2021). The impact of seropositivity on the effectiveness of biologic anti-rheumatic agents: Results from a collaboration of 16 registries. Rheumatology.

[3] Iannone F., Gremese E., Atzeni F., Biasi D., Botsios C., Cipriani P., Ferraccioli G., Lapadula G., Salaffi F., Triolo G. (2012). Long-term retention of tumor necrosis factor-inhibitor therapy in a large italian cohort of patients with rheumatoid arthritis from the GISEA registry: An appraisal of predictors. J. Rheumatol..

[4] Monti S., Grosso V., Todoerti M., Caporali R. (2018). Randomized controlled trials and real-world data: Differences and similarities to untangle literature data. Rheumatology.

[5] Brevet P., Fréret M., Barat E., Carvajal Alegria G., Cornec D., Saraux A., Devauchelle-Pensec V. (2025). Could the anti-modified protein antibody concept help better define seronegative rheumatoid arthritis?. Jt. Bone Spine.

[6] Svensson B., Andersson M.L.E., Gjertsson I., Hafström I., Forslind K. (2022). Erosion-free rheumatoid arthritis: Clinical and conceptional implications—A BARFOT study. BMC Rheumatol..

[7] Van der Heijde D. (2000). How to read radiographs according to the Sharp/van der Heijde method. J. Rheumatol..

[8] Colebatch A.N., Edwards C.J., Østergaard M., van der Heijde D., Balint P.V., D’Agostino M.-A., Forslind K., Grassi W., Haavardsholm E.A., Haugeberg G. (2013). EULAR Recommendations for the Use of Imaging of the Joints in the Clinical Management of Rheumatoid Arthritis. Ann. Rheum. Dis..

[9] Chevet B., Cornec D. (2025). Clinical presentation and treatment response in ACPA-negative rheumatoid arthritis. Jt. Bone Spine.

[10] Sahatçiu-Meka V., Rexhepi S., Manxhuka-Kërliu S., Rexhepi M. (2010). Extra-articular manifestations of seronegative and seropositive rheumatoid arthritis. Bosn. J. Basic Med. Sci..

[11] Matthijssen X.M.E., Huizinga T.W.J., van der Helm-van Mil A.H.M. (2022). Increasing incidence of autoantibody-negative RA is replicated and is partly explained by an aging population. Ann. Rheum. Dis..

[12] Myasoedova E., Davis J.M., Matteson E.L., Crowson C.S. (2020). Is the epidemiology of rheumatoid arthritis changing? Results from a population-based incidence study, 1985–2014. Ann. Rheum. Dis..

[13] Konig M.F., Abusleme L., Reinber J., Mandal P., Konstantinidis A., Archontis A., Mit-Lin N., Arguello F., Fennelly G.J., Goh S.G. (2016). *Aggregatibacter actinomycetemcomitans*-induced hypercitrullination links periodontal infection to autoimmunity in rheumatoid arthritis. Sci. Transl. Med..

[14] Graves D.T., Corrêa J.D., Silva T.A. (2019). The oral microbiota is modified by systemic diseases. J. Dent. Res..

[15] De Stefano L., D’Onofrio B., Manzo A., Sakellariou G., Bugatti S., Montecucco C. (2021). The genetic, environmental, and immunopathological complexity of autoantibody-negative rheumatoid arthritis. Int. J. Mol. Sci..

[16] Chang C., Lu Q. (2022). MicroRNA-mediated epigenetic regulation of rheumatoid arthritis. Front. Immunol..

[17] De Stefano L., Bugatti S., Mazzucchelli I., Rossi S., Xoxi B., Bozzalla Cassione E., Delvino P., Sakellariou G., Bugatti A., Manzo A. (2024). Synovial and serum B-cell signature of autoantibody-negative rheumatoid arthritis versus autoantibody-positive rheumatoid arthritis and psoriatic arthritis. Rheumatology.

[18] Wu X., Liu Y., Jin S., Wang M., Jiao Y., Yang H., Chen B., Cai R., Lu Y., Zhao S. (2021). Single-cell sequencing of immune cells from anticitrullinated peptide antibody–positive and –negative rheumatoid arthritis. Nat. Commun..

[19] Pap T., Müller-Ladner U., Gay R.E., Gay S. (2000). Fibroblast biology: Role of synovial fibroblasts in the pathogenesis of rheumatoid arthritis. Arthritis Res. Ther..

[20] Shi J., van de Stadt L.A., Levarht E.W.N., Huizinga T.W.J., Toes R.E.M., Trouw L.A., Van Schaardenburg D. (2013). Anti-Carbamylated Protein Antibodies Are Present in Arthralgia Patients and Predict the Development of Rheumatoid Arthritis. Arthritis Rheum..

[21] Boeters D.M., Trouw L.A., van der Helm-van Mil A.H.M. (2018). Does information on novel identified autoantibodies contribute to predicting the progression from undifferentiated arthritis to rheumatoid arthritis: A study on anti-CarP antibodies as an example. Arthritis Res. Ther..

[22] Nijjar J.S., Morton F.R., Bang H., Buckley C.D., van der Heijde D., Gilmour A., Paterson C., McInnes I.B., Porter D., Raza K. (2021). Scottish Early Rheumatoid Arthritis Inception Cohort Investigators.. The impact of autoantibodies against citrullinated, carbamylated, and acetylated peptides on radiographic progression in patients with new-onset rheumatoid arthritis: An observational cohort study. Lancet Rheumatol..

[23] Dibrov D.A., Avdeeva A.S., Diatroptov M.E., Nasonov E.L. (2024). Anti-Carbamylated Protein Antibodies in ACPA-Negative and ACPA-Positive Patients with Rheumatoid Arthritis. Dokl. Biochem. Biophys..

[24] Zhang G., Xu J., Du D., Gao L., Gan F., Zhang X., Wang J., Xu Y., Chen Y. (2023). Diagnostic values, association with disease activity and possible risk factors of anti-PAD4 in rheumatoid arthritis: A meta-analysis. Rheumatology.

[25] Ren J., Sun L., Zhao J. (2017). Meta-analysis: Diagnostic accuracy of antibody against peptidylarginine deiminase 4 by ELISA for rheumatoid arthritis. Clin. Rheumatol..

[26] Yi Y., Lei L., Sun Y., Mei J., Zhang Y., Chen J., Ying G., Wu Y. (2025). Biomarkers for early diagnosis of rheumatoid arthritis. Clin. Chim. Acta.

[27] 27, Floris A., Angioni M.M., Fadda M., Naitza M.R., Congia M., Chessa E., Piga M., Cauli A. (2025). The role of anti-PAD4, anti-CarP, and anti-RA33 antibodies combined with RF and ACPA in predicting abatacept response in rheumatoid arthritis. Arthritis Res. Ther..

[28] Lukas C., Combe B., Ravaud P., Sibilia J., Landewé R., van der Heijde D. (2011). Favorable effect of very early disease-modifying antirheumatic drug treatment on radiographic progression in early inflammatory arthritis: Data from the Étude et Suivi des Polyarthrites IndifféRenciées récentes (Study and Followup of Early Undifferentiated Polyarthritis.). Arthritis Rheum..

[29] Smolen J.S., Landewé R.B.M., Bijlsma J.W.J., Burmester G.R., Dougados M., Kerschbaumer A., McInnes I.B., Sepriano A., van Vollenhoven R.F., de Wit M. (2020). EULAR recommendations for the management of rheumatoid arthritis with synthetic and biological disease-modifying antirheumatic drugs: 2019 update. Ann. Rheum. Dis..

[30] Aletaha D., Neogi T., Silman A.J., Funovits J., Felson D.T., Bingham C.O., Birnbaum N.S., Burmester G.R., Bykerk V.P., Cohen M.D. (2010). 2010 Rheumatoid arthritis classification criteria: An American College of Rheumatology/European League Against Rheumatism collaborative initiative. Ann. Rheum. Dis..

[31] Sparks J.A. (2017). Rheumatoid arthritis classification criteria: Updated perspectives. Rheum. Dis. Clin. N. Am..

[32] Van der Helm-van Mil A.H.M., le Cessie S., van Dongen H., Breedveld F.C., Toes R.E.M., Huizinga T.W.J. (2007). A prediction rule for disease outcome in patients with recent-onset undifferentiated arthritis: How to guide individual treatment decisions. Arthritis Rheum..

[33] Van Steenbergen H.W., Huizinga T.W.J., van der Helm-van Mil A.H.M. (2015). Clinical and serological factors associated with radiographic progression in early arthritis: A meta-analysis. Ann. Rheum. Dis..

[34] Machold K.P., Stamm T.A., Eberl G.J., Nell V.K., Dunky A., Uffmann M., et Smolen J.S. (2002). Very recent onset arthritis—Clinical, laboratory, and radiological findings during the first year of disease. J. Rheumatol..

[35] Pincus T., Callahan L.F., Sale W.G., Brooks A.L., Payne L.E., Vaughn W.K. (1998). Rheumatoid arthritis subgroups: Patients with negative rheumatoid factor in long-term clinical studies. J. Rheumatol..

[36] Nordberg L.B., Lillegraven S., Lie E., Aga A.B., Olsen I.C., Hammer H.B., Uhlig T., Jonsson M.K., van der Heijde D., Kvien T.K. (2017). Patients with seronegative RA have more inflammatory activity compared with patients with seropositive RA in an inception cohort of DMARD-naïve patients classified according to the 2010 ACR/EULAR criteria. Ann. Rheum. Dis..

[37] Rönnelid J., Wick M.C., Lampa J., Lindblad S., Nordmark B., Klareskog L., van Vollenhoven R.F. (2005). Longitudinal analysis of citrullinated protein/peptide antibodies (anti-CP) during 5 year follow up in early rheumatoid arthritis: Anti-CP status predicts worse disease activity and greater radiological progression. Ann. Rheum. Dis..

[38] Barra L., Pope J.E., Orav J.E., Engel E., Lee E., Engel P.J., Engel G., Engel C. (2014). Prognosis of seronegative patients in a large prospective cohort of patients with early inflammatory arthritis. J. Rheumatol..

[39] Boeters D.M., Gaujoux-Viala C., Constantin A., van der Helm-van Mil A.H.M. (2017). The 2010 ACR/EULAR criteria are not sufficiently accurate in the early identification of autoantibody-negative rheumatoid arthritis: Results from the Leiden-EAC and ESPOIR cohorts. Semin. Arthritis Rheum..

[40] Van der Heijde D., van der Helm-van Mil A.H.M., Aletaha D., Bingham C.O., Burmester G.R., Dougados M., Emery P., Felson D., Knevel R., Kvien T.K. (2013). EULAR definition of erosive disease in light of the 2010 ACR/EULAR rheumatoid arthritis classification criteria. Ann. Rheum. Dis..

[41] Brown A.K., Conaghan P.G., Karim Z., Quinn M.A., Ikeda K., Peterfy C.G., Hensor E.M.A., Wakefield R.J., O’Connor P.J., Emery P. (2008). An explanation for the apparent dissociation between clinical remission and continued structural deterioration in rheumatoid arthritis. Arthritis Res. Ther..

[42] Hammer H.B., Hausmann J.S., Østergaard M. (2014). Imaging in early rheumatoid arthritis—What is the best method for detecting erosions and disease progression?. Curr. Opin. Rheumatol..

[43] Wu S., Griffith J.F., Xiao F., Yiu C., Leung J.C.S., Tam L.S. (2025). Early rheumatoid arthritis, two distinctive structural damage patterns revealed by MRI: An 8-year longitudinal study. Eur. Radiol..

[44] Mo Q., Wang M., Cai S., Zhong J., Dong L. (2024). A comparative study on the clinical and magnetic resonance imaging features between seronegative and seropositive rheumatoid arthritis. Clin. Exp. Rheumatol..

[45] Wang J., Wang M., Qi Q., Wu Z., Wen J. (2022). High-frequency ultrasound in patients with seronegative rheumatoid arthritis. Sci. Rep..

[46] Cen Y., He D., Wang P., Qin Y., Huang M. (2024). Contribution of Musculoskeletal Ultrasound in the Diagnosis of Seronegative Rheumatoid Arthritis. J. Ultrasound Med..

[47] 47, Xu J., Gong Y., Yang K., Fang Y., Li W., Chen S. (2024). A cohort study of ultrasonic semi-quantitative scoring for the diagnosis of serology-negative rheumatoid arthritis. Arch. Rheumatol..

[48] Gossec L., Paternotte S., Combe B., Meyer O., Dougados M. (2014). 2014 (ESPOIR) Repeated anticitrullinated protein antibody and rheumatoid factor assessment is not necessary in early arthritis. J. Rheumatol..

[49] Tenstad E., Andreassen M.L., Thoen J. (2020). Use and utility of serologic tests for rheumatoid arthritis in primary care. Dan. Med. J..

[50] Hiwa M., Seto T., Ueno Y., Nagasawa H., Minota S. (2017). Only rheumatoid factor-positive subset of ACPA-negative RA may seroconvert to ACPA-positive. Int. J. Rheum. Dis..

[51] Reid J., Gorelik A., Engelbrecht L., Gayed S.L., Rischmueller M. (2020). Repeat serological testing for ACPAs after diagnosis is not useful. Intern. Med. J..

[52] Coffey C.M., Crowson C.S., Myasoedova E., Matteson E.L., Davis J.M. (2019). Evidence of diagnostic and treatment delay in seronegative rheumatoid arthritis: Missing the window of opportunity. Mayo Clin. Proc..

[53] Paalanen K., Rannio K., Rannio T., Asikainen J., Hannonen P., Sokka T. (2020). Prevalence of calcium pyrophosphate deposition disease in a cohort of patients diagnosed with seronegative rheumatoid arthritis. Clin. Exp. Rheumatol..

[54] Krekeler M., Baraliakos X., Tsiami S., Braun J. (2022). High prevalence of chondrocalcinosis and frequent comorbidity with calcium pyrophosphate deposition disease in patients with seronegative rheumatoid arthritis. RMD Open.

[55] Bugatti S., De Stefano L., Gandolfo S., Ciccia F., Montecucco C. (2023). Autoantibody-negative rheumatoid arthritis: Still a challenge for the rheumatologist. Lancet Rheumatol..

[56] Mahalingam S., Herrero L.J., Taylor A., Morse L.P., Lidbury B.A., Rolph M.S., Mahalingam S., Herrero L.J., Herring B.L. (2021). Viral arthritis. Arthropod Borne Diseases.

[57] Portillo D.C., Puéchal X., Masson M., Kostine M., Michaut A., Ramon A., Wendling D., Costedoat-Chalumeau N., Richette P., Marotte H. (2024). Diagnosis and treatment of *Tropheryma whipplei* infection in patients with inflammatory rheumatic disease: Data from the French Tw-IRD registry. J Infect..

[58] Caporali R., Montecucco C., Epis O., Bobbio-Pallavicini F., Maio T., Cimmino M.A. (2001). Presenting features of polymyalgia rheumatica (PMR) and rheumatoid arthritis with PMR-like onset: A prospective study. Ann. Rheum. Dis..

[59] Shen J.Z., Carlsen E.D., Carrillo L.F., Cintron D., Kellogg B., Lim J., Nicholas M., Lackey E., Dasher J. (2025). Lymphoproliferative Disorders Mimicking Rheumatologic Disease: A Clinical Reasoning Perspective. Curr. Allergy Asthma Rep..

[60] Al Khayyat S.G., Conticini E., Falsetti P., Gentileschi S., Vitale A., Stella S.M., D’Alessandro M., Bargagli E., Cantarini L., Frediani B. (2022). Paraneoplastic arthritides: An up-to-date case-based systematic review. Reumatologia.

[61] Lewik G., Müller L.S., Lewik G. (2023). First case report of rheumatoid-like paraneoplastic polyarthritis in a patient with Fallopian tube cancer. Rheumatol. Adv. Pract..

[62] Braaten T.J., Brahmer J.R., Forde P.M., Le D., Lipson E.J., Naidoo J., Schollenberger M., Zheng L., Bingham C.O., Shah A.A. (2020). Immune checkpoint inhibitor-induced inflammatory arthritis persists after immunotherapy cessation. Ann. Rheum. Dis..

[63] Bernabela L., Bermas B. (2024). Immune checkpoint inhibitor associated rheumatoid arthritis. Curr. Rheumatol. Rep..

[64] Cappelli L.C., Bingham C.O., Braaten T., Shah A.A. (2022). Response to: Correspondence on “Immune checkpoint inhibitor-induced inflammatory arthritis persists after immunotherapy cessation” by Braaten et al. Ann. Rheum. Dis..

[65] McCarter K.R., Wolfgang T., Arabelovic S., Wang X., Yoshida K., Banasiak E.P., Qian G., Kowalski E.N., Vanni K.M.M., LeBoeuf N.R. (2023). Mortality and immune-related adverse events after immune checkpoint inhibitor initiation for cancer among patients with pre-existing rheumatoid arthritis: A retrospective, comparative cohort study. Lancet Rheumatol..

[66] Zhao M., Mi L., Ji Y., He X., Gao Y., Hu Y., Xu K. (2023). Advances of autoimmune rheumatic diseases related to malignant tumors. Inflamm. Res..

[67] Witthoek R., Carron P., Verbruggen G. (2023). Erosive and non-erosive hand osteoarthritis: An update on diagnosis and management. RMD Open.

[68] Paalanen K., Rannio K., Rannio T., Asikainen J., Hannonen P., Sokka T. (2019). Does early seronegative arthritis develop into rheumatoid arthritis? A 10-year observational study. Clin. Exp. Rheumatol..

[69] Schett G., Gravallese E. (2012). Bone erosion in rheumatoid arthritis: Mechanisms, diagnosis and treatment. Nat. Rev. Rheumatol..

[70] Henderson B., Goldring S.R. (2015). Mechanisms of bone loss in inflammatory arthritis. Nat Rev Rheumatol..

[71] Jilani A.A., Mackworth-Young C.G. (2015). The role of citrullinated protein antibodies in predicting erosive disease in rheumatoid arthritis: A systematic literature review and meta-analysis. Int. J. Rheumatol..

[72] Harre U., Georgess D., Bang H., Bozec A., Axmann R., Ossipova E., Jakobsson P.J., Baber W., Snir O., Makrygiannakis D. (2012). Induction of osteoclastogenesis and bone loss by human autoantibodies against citrullinated vimentin. J. Clin. Investig..

[73] van der Heijde D., van der Helm-van Mil A.H.M., Aletaha D., Bukhari M., Landewe R., Machado P.M. (2013). Erosion progression in rheumatoid arthritis: Relationship with disease activity and autoantibody status. Ann. Rheum Dis..

[74] Grosse J., Allado E., Roux C., Pierreisnard A., Couderc M., Clerc-Urmes I., Claudel J., Chary-Valckenaere I., Loeuille D. (2020). ACPA-positive versus ACPA-negative rheumatoid arthritis: Two distinct erosive disease entities on radiography and ultrasonography. Rheumatol. Int..

[75] Gadeholt O., Hausotter K., Eberle H., Klink T., Pfeil A. (2019). Differing X-ray patterns in seronegative and seropositive rheumatoid arthritis. Clin. Rheumatol..

[76] Liao K.P., Weinblatt M.E., Cui J., Iannaccone C., Chibnik L.B., Lu B., Iannaccone C., Chibnik L.B., Lu B., Engel E. (2011). Clinical predictors of erosion-free status in rheumatoid arthritis: A prospective cohort study. Rheumatology.

[77] Tobón G., Saraux A., Lukas C., Gandjbakhch F., Gottenberg J.E., Mariette X., Gandjbakhch F., Gottenberg J.E., Mariette X., Fautrel B. (2013). First-year radiographic progression as a predictor of further progression in early arthritis: Results of a large national French cohort. Arthritis Care Res..

[78] D’Onofrio B., Selmi C., Gremese E. (2025). Are seronegative patients with rheumatoid arthritis and clinically suspect arthralgia properly represented in randomized clinical trials?. Clin. Rheumatol..

[79] van Nies J.A.B., Tsonaka R., Gaujoux-Viala C., Fautrel B., van der Helm-van Mil A.H.M. (2015). Evaluating relationships between symptom duration and persistence of rheumatoid arthritis: Does a window of opportunity exist? Results on the Leiden Early Arthritis Clinic and ESPOIR cohorts. Ann. Rheum. Dis..

[80] Javed I., Crowson C.S. (2025). The apprehension of seronegative rheumatoid arthritis. Nat. Rev. Rheumatol..

[81] Boers M., Hartman L., Opris-Belinski D., Bos R., Kok M.R., Da Silva J.A.P., Griep E.N., Klaasen R., Allaart C.F., Baudoin P. (2022). Low-dose, add-on prednisolone in patients with rheumatoid arthritis aged 65+: The pragmatic randomised, double-blind placebo-controlled GLORIA trial. Ann. Rheum. Dis..

[82] Lukas C., Mary J., Debandt M., Daïen C., Morel J., Cantagrel A., Fautrel B., Combe B. (2019). Predictors of good response to conventional synthetic DMARDs in early seronegative rheumatoid arthritis: Data from the ESPOIR cohort. Arthritis Res. Ther..

[83] Duong S.Q., Crowson C.S., Athreya A., Atkinson E.J., Davis J.M., Warrington K.J., Matteson E.L., Weinshilboum R., Wang L., Myasoedova E. (2022). Clinical predictors of response to methotrexate in patients with rheumatoid arthritis: A machine learning approach using clinical trial data. Arthritis Res. Ther..

[84] Sokolove J., Schiff M., Fleischmann R., Weinblatt M.E., Connolly S.E., Johnsen A., Zhu J., Maldonado M.A., Engel G., Lee E. (2016). Impact of baseline anti-cyclic citrullinated peptide-2 antibody concentration on efficacy outcomes following treatment with subcutaneous abatacept or adalimumab: 2-year results from the AMPLE trial. Ann. Rheum. Dis..

[85] Harrold L.R., Connolly S.E., Wittstock K., Guyette E., Engel E., Engel P., Engel G., Engel C., Engel P.J., Lee E. (2022). Baseline anti-citrullinated protein antibody status and response to abatacept or non-TNFi biologic/targeted synthetic DMARDs: US observational study of patients with RA. Rheumatol. Ther..

[86] Kida D., Takahashi N., Kaneko A., Hirano Y., Ogawa T., Fujibayashi T., Hanabayashi M., Hattori Y., Noda K., Miyake H. (2020). A retrospective analysis of the relationship between anti-cyclic citrullinated peptide antibody and the effectiveness of abatacept in rheumatoid arthritis patients. Sci. Rep..

[87] Lin C.-T., Huang W.-N., Tsai W.-C., Chen J.P., Wei J.C.C., Chen C.H. (2021). Predictors of drug survival for biologic and targeted synthetic DMARDs in rheumatoid arthritis. PLoS ONE.

[88] Bird P., Hall S., Nash P., Connell C.A., Kwok K., Witcombe D., Thirunavukkarasu K. (2019). Treatment outcomes in patients with seropositive versus seronegative rheumatoid arthritis in phase III randomized clinical trials of tofacitinib. RMD Open.

[89] Shipa M.R.A., Di Cicco M., Balogh E., Nitu N.A., Mainuddin M.D., Bhadauria N., Mukerjee D., Roussou E. (2023). Drug-survival profiling of second-line biologic therapy in rheumatoid arthritis: Choice of another tumour necrosis factor inhibitor or a biologic of different mode of action?. Mod. Rheumatol..

[90] Boyapati A., Msihid J., Fiore S., van Adelsberg J., Graham N.M.H., Hamilton J.D. (2016). Sarilumab plus methotrexate suppresses circulating biomarkers of bone resorption and synovial damage in patients with rheumatoid arthritis and inadequate response to methotrexate: A biomarker study of MOBILITY. Arthritis Res. Ther..

[91] Yokota K., Miyazaki T., Sato K., Tanaka Y. (2021). TNF- and IL-6-dependent osteoclastogenesis independent of RANKL via activation of NFATc1 and JAK signaling in inflammatory arthritis. Int Immunol..

[92] Chung S.W. (2025). Promising molecular therapeutic targets for drug development in rheumatoid arthritis. J. Clin. Med..

[93] Arneson L.C., Carroll K.J., Ruderman E.M. (2021). Bruton’s tyrosine kinase inhibition for the treatment of rheumatoid arthritis. ImmunoTargets Ther..

